# Tiller-specific formulated fertilizer improves the population tiller quantity and yield of machine-transplanted rice

**DOI:** 10.1371/journal.pone.0345537

**Published:** 2026-03-25

**Authors:** Jun Deng, Xuehuan Liao, Jun Shi, Qi Xu, Xinyu Yang, Ran Wang, Jianming Ding, Lubing Jia, Xiaotian Jiang, Shengmin Yan, Rongping Zhang, Yungao Hu, Peng Ma

**Affiliations:** 1 College of Life Sciences and Agri-forestry, Southwest University of Science and Technology, Mianyang, China; 2 Crop Germplasm Innovation and Genetic Improvement Key Laboratory of Sichuan Province/ Mianyang Academy of Agricultural Sciences, Mianyang, China; 3 Zigong Academy of Agricultural Sciences, Zigong, China; Nepal Agricultural Research Council, NEPAL

## Abstract

**Abstract** Delayed harvest of the previous crops of the rice-based rotation systems in Sichuan often leads to late transplanting of rice, thus extending the seedling age and shortening the effective growth period after the recovery of machine-transplanted seedlings. To improve the seedling survival rate and root growth after mechanical transplantation, as well as to promote tillering and regreening, a two-factor split-plot experiment was conducted. The main plot factor was the application of tiller fertilizer, and the subplot factor was the rice variety. Two fertilizer treatments were established, namely compound fertilizer (CK) and formula-specific tiller fertilizer (T1), where CK was used as the control. Four hybrid rice varieties were Longliangyou 534 (V1), Yunliangyou 332 (V2), Taifengyou 208 (V3), and Nei 6 You 6368 (V4). This study investigated the effects of the formulaic tiller fertilizer on the tillering rate, growth, development, and yield of machine-transplanted hybrid rice. The results showed that the effective panicle rate of rice increased by 1.26% under the T1 treatment compared to that under CK. Ten days after tillering, the length and fresh weight of tiller buds were 53.65% and 67.06% notably higher in T1 than those in CK. Additionally, the bud length was peaked in V4. Compared with CK, T1 treatment significantly increased the content of auxin (IAA), endogenous zeatin + zeatin riboside (Z + ZR), and the ethylene precursor 1-aminocyclopropane-1-carboxylic acid (ACC) in tiller buds, while reducing the content of abscisic acid (ABA). Additionally, the formula-based tiller fertilizer remarkably enhanced the activity of Ca² ⁺ -Mg² ⁺ -ATPase. Compared to CK, the yield contributions of the main stem, primary tillers, and secondary tillers under treatment T1 increased by 6.96%, 18.42%, and 8.86% in 2022, respectively. Among all treatments, the V3T1 treatment resulted in the highest yields of the main stem and primary tillers, with yield contribution rates of 9.79% and 56.63%, respectively. In 2023, the yields of the main stem, primary tillers, and secondary tillers under T1 were higher than those under CK. Specifically, T1 had the highest yield contribution rate from primary tillers, whereas the contribution rate of secondary tillers was lower than that of CK. For the V4 growth stage, T1 also produced the highest yields of the main stem, primary tillers, and secondary tillers, at 988.49 kg/hm², 5,432.55 kg/hm², and 5,050.10 kg/hm², respectively. Therefore, formula-specific tiller fertilizer(T1) exhibited a significant yield-increasing effect on hybrid rice, with greater yield potential observed in V3 and V4. These findings provide a technical reference for mechanized transplantation cultivation of rice in the hilly rice-growing areas of northwestern Sichuan.

## 1. Introduction

Rice is the largest and highest-yielding staple crop in Sichuan, accounting for more than 45% of the cropping area in the southwest rice region and contributing to more than 50% of the region’s rice output, thus playing a critical role in national food security [1]. Rapeseed/wheat-rice rotation is the dominant cropping pattern in the hilly rice-growing areas of northwestern Sichuan. However, frequent early summer droughts in these areas, coupled with delayed harvest of rapeseed and wheat, result in late transplanting of rice, prolonged seedling age, poor seedling quality and an extended regreening period of machine-transplanted rice seedlings, all of which impair the transplanting quality and yield formation of rice. [2]. In practice, farmers blind pursuit for high yielding of rice by excessive chemical nitrogen and compound fertilizers inputs resulting in severe non-point source pollution and reduced fertilizer use efficiency. Tillering development, a key determinant of rice yield, is governed by genetic characteristics, phytohormones, and environmental factors [3]; activation of tiller primordia is regulated by endogenous hormone levels, with cytokinin concentration being a critical trigger for bud germination. Further growth of tiller buds depends on nutrient supply, and these nutritional factors collectively regulate the initiation and development of rice tillers [4]. In early tillering, plants show a coordinated pattern of rising zeatin levels and declining gibberellin (GA3) and auxin (IAA) concentrations; GA3, together with IAA and cytokinins (CTKs), modulates tillering through both synergistic and antagonistic interactions [5]. Elevated endogenous zeatin and zeatin riboside (Z + ZR) concentrations are correlated with greater tiller-bud fresh weight and length [6]. Given the complexity of the hormonal regulation of lateral bud growth, no definitive model currently exists to fully explain the relationship between phytohormones and tillering [7,8].

The medium and trace elements(Si, Mg, Zn etc.)are crucial for the growth and development of rice. They participate in plant physiological metabolism and enzymatic reactions, regulate key agronomic processes such as tiller germination and panicle development, significantly improve yield components, enhance rice stress resistance, and reduce the incidence of pests and diseases, thereby increasing rice yield and improving grain quality [9]. Studies have shown that zinc can promote the synthesis of tryptophan, the precursor of indole-3-acetic acid (IAA), and up-regulate the expression of cytokinin (CTK) synthetic genes, thereby increasing the CTK/IAA ratio and breaking the dormancy of tiller buds. It also activates cell division proteins in the meristems of tiller buds, enhances cell division activity, and increases the number of tiller primordia. Elements such as manganese and molybdenum participate in ATP synthesis and energy metabolism, providing sufficient energy for the rapid division of meristems, accelerating the early occurrence of tillering after the regreening of late-transplanted rice seedlings, alleviating hormone imbalance and energy deficiency caused by transplanting stress, and significantly improving the tillering rate and productive tiller percentage [10,11]. Studies have indicated that supplementing conventional fertilization with Si and Zn significantly increases grain yield by increasing panicle number per unit area and grains per panicle [12,13].

Current research on rice production largely focuses on placement methods, tiller fertilizer management, reducing chemical fertilizer rates, and increasing organic or micronutrient inputs to achieve balanced fertilization and improve crop yield and quality [14–16]. However, these approaches do not fully address problems induced by delayed harvests of rapeseed, wheat, or other cash crops-namely late transplanting, extended seedling age, poor seedling quality, prolonged greening of machine-transplanted seedlings and reduced tiller initiation. In this study, micronutrient fertilizers were incorporated into conventional compound fertilizers to investigate the effects and action mechanisms of micronutrients on the balance of cytokinins and auxins, tiller bud germination, enhancement of energy supply to meristems, promotion of seedling regreening, and acceleration of early and vigorous tillering. Additionally, the impacts of the formulated tiller fertilizer on the population structure and grain yield of machine-transplanted rice were studied, aiming to provide a theoretical basis for the rational combined application of fertilizers in machine-transplanted rice cultivation.

## 2. Materials and methods

### 2.1. Experimental materials

Longliangyou 534 (National Approved Rice 20170001): an indica two-line hybrid rice variety, jointly bred by Yuan Longping High-Tech Agriculture Co., Ltd., Rice Research Institute of Guangdong Academy of Agricultural Sciences, Shenzhen Longping Jingu Seed Industry Co., Ltd., and Hunan Yahua Seed Industry Academy of Agricultural Sciences. It has a total growth period of 158.5 days, with the parental combination of Longke 638S × R534.

Yunliangyou 332 (National Approved Rice 20196008): an indica two-line hybrid rice variety, jointly bred by Hunan Longping Seed Industry Co., Ltd. and Shenzhen Zhaonong Agricultural Technology Co., Ltd. It has a total growth period of 132.2 days, with the parental combination of Yun 2013S × W332.

Taifengyou 208 (Guangdong Approved Rice 2012031): a temperature-sensitive three-line hybrid rice variety, bred by the Rice Research Institute of Guangdong Academy of Agricultural Sciences. It has a total growth period of 110–112 days, with the parental combination of Taifeng A × Guanghui 208.

Nei 6you 6368 (Guizhou Approved Rice 20180017): an indica three-line hybrid rice variety, bred by Sichuan Fasheng Rice Technology Co., Ltd. It has a total growth period of 155.8 days, with the parental combination of Neixiang 6A × Jianghui 4368.

All the four varieties possess strong tillering ability, a large number of productive panicles and grains per panicle, as well as a high seed-setting rate, and exhibit a high yield level when grown as single-season mid-season rice in the upper reaches of the Yangtze River. The rice-specific compound fertilizer used was “Jinzhengda 3+” (N-P_2_O_5_-K₂O: 18-12-10), produced by the Kingenta (Jinzhengda) Group. The formulated tiller fertilizer consisted of multiple micro-and trace elements (Si, Mg, and Zn) applied at a rate of 1 kg/667 m², Fe + Zn + B + Mo + Cu + Mu ≥ 10.0%, Si ≥ 2.20%, MgO ≥ 9.0%. It was manufactured by Shandong Shitianbu Agricultural Development Co., Ltd.

### 2.2. Experimental design

**Fig 1:**
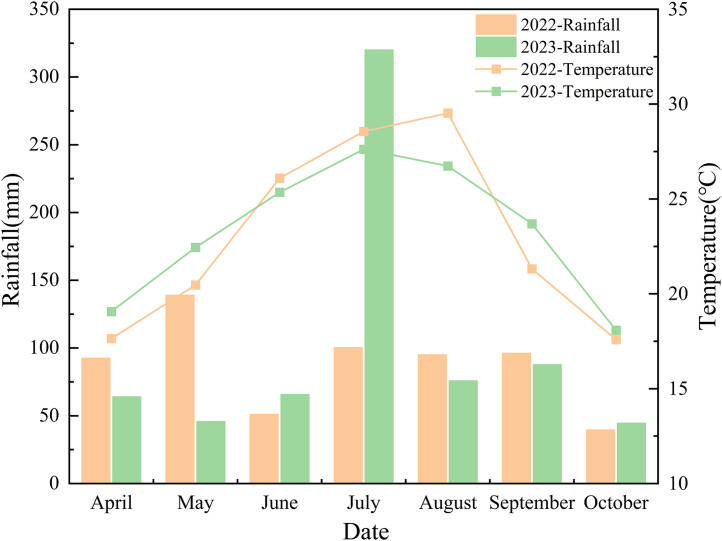
Meteorological data of temperature and precipitation in the experimental area.

The experiment was conducted from 2022–2023 at the Experimental Farm of Southwest University of Science and Technology, located at 31°53′N latitude and 104°70′E longitude. The experimental site had medium-to-high soil fertility and was previously used as a winter fallow field. The physicochemical properties of the plowed soil layer (0–20 cm) were as follows: total nitrogen (N)1.65 g/kg, total phosphorus (P)0.73 g/kg, total potassium (K)1.55 g/kg, and pH 7.10. A two-factor split-plot experimental design was adopted, with tiller fertilizer application as the main plot factor, consisting of two treatments: compound fertilizer (CK, control) and compound fertilizer + formulated tiller fertilizer (T1). The subplot factors included the four hybrid rice varieties. Each treatment was replicated thrice. Seedlings were raised in substrate-filled trays on April 26 at a seeding rate of 80 g per tray. Machine transplantation was implemented on May 28 at a planting density of 36 × 18 cm, and each subplot had an area of 40 m². For the CK treatment, compound fertilizer was applied at a total N rate of 150 kg/hm², following a split application ratio of base fertilizer:tiller fertilizer:panicle fertilizer of 5:3:2. The formulated tiller fertilizer was thoroughly mixed with the compound fertilizer component and broadcast uniformly for 7 d after transplanting. All other agronomic management measures were consistent with the local high-yield cultivation practices. Fig 1. shows the meteorological data collected during the trial (sourced from the Mianyang Meteorological Station, Sichuan Province).

### 2.3. Measurement indicators and methods

#### 2.3.1. Assessment of panicle formation rate (tiller-to-panicle conversion).

At early tillering, 30 healthy, pest-free, and disease-free plants, whose tiller counts matched the plot mean, were tagged. The number of tillers per plant was monitored over time until it stabilized. The maximum tiller number and number of effective panicles at maturity were recorded to calculate the tiller-to-panicle conversion rate.


Tiller−to−panicle conversion rate (%) = (effective panicle number/maximum tiller number) ×100

#### 2.3.2. Endogenous hormone content of tiller buds.

Beginning at maximum tillering, tiller-bud growth was monitored at 5-d intervals; for each treatment, three plants were sampled, leaves and sheaths were removed and nodal tiller buds were excised, with ten buds pooled per sample (fresh weight averaged). Additionally, bud length was measured. Samples were flash-frozen in liquid nitrogen and stored at −80°C. Hormone concentrations (ABA, IAA, Z + ZR, and ACC) in the tiller-bud tissue were quantified by HPLC (Waters 2695) according to Yang et al [17].

#### 2.3.3. Measurement of Ca²^+^-Mg²^+^-ATPase activity.

From the full-heading stage, flag leaves were sampled from different plants at 5-d intervals. Collected leaves were wrapped in aluminum foil, flash-frozen in liquid nitrogen and transferred to a −80°C ultra-low freezer for storage until analysis. Ca^2+^–Mg^2+^–ATPase activity was assayed as described by Datiles et al [18].

#### 2.3.4. Yield and yield components.

At maturity, five plants per subplot were sampled based on the mean effective panicle number. Seed counting was performed separately for the main stem, first-order, and second-order tillers, and grain yield was calculated from 15 plants using the average effective panicle number. Seed traits (number of filled grains, number of empty grains, and thousand grain weight) were measured using a Wanshen automatic seed-testing analyzer (model SC-G), and the effective panicle number, grains per panicle, and seed-set rate were subsequently calculated.

### 2.4. Data processing and analysis

Data were organized and analyzed using Microsoft Excel 2016. Further statistical analyses were conducted using DPS 7.05, and multiple comparisons among treatments were performed using the least significant difference (LSD) test. Figures were generated using Origin 2021.

## 3. Results

### 3.1. Effect of formulated tillering fertilizer on rice tiller-to-panicle formation rate

**Fig 2:**
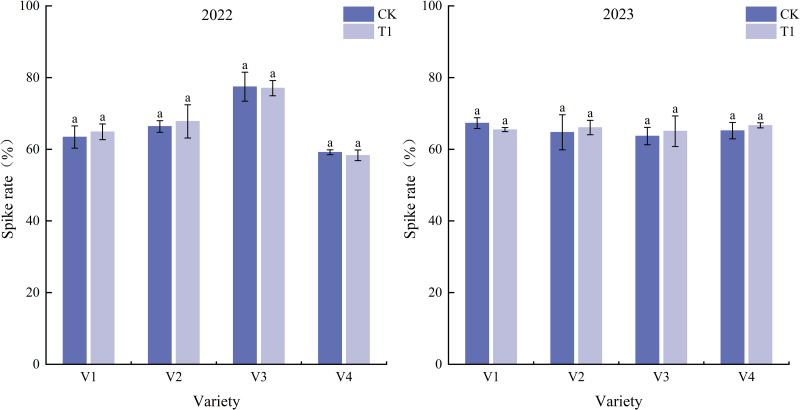
Tiller-bearing Panicle Rate of Rice Under Different Tiller Fertilizer Treatments. Note: Different letters indicate significant differences (*p* < 0.05) among treatments within the same variety; the same notation is used hereafter.

The effects of formulated tiller fertilizer on the tiller-to-panicle formation rate are presented in Fig 2. In 2022, no significant differences in the tiller-to-panicle formation rate were detected among different fertilizer treatments. Under T1, the tiller-to-panicle formation rate slightly decreased in varieties V3 and V4, while it increased by 2.32% and 2.15% in V1 and V2, respectively, compared with CK. Among all the tested varieties, the tiller-to-panicle formation rate of V3 exceeded 77%, which was significantly higher than that of the other three varieties. In 2023, the T1 treatment generally improved the tiller-to-panicle formation rate, and no significant differences were observed among the four varieties. Notably, the tiller-to-panicle formation rate of V1 under the CK treatment was slightly lower than that of the other varieties. For V2, V3, and V4, the T1 treatment resulted in higher tiller-to-panicle formation rates than the CK treatment.

A comparison between years revealed that in 2022, the increase in T1 relative to CK ranged from 3.2% to 5.1%, with V3 showing the largest increase; in 2023, the increase in T1 relative to CK ranged from 2.8% to 4.5%, and the overall increase was slightly lower than that in 2022. V3 exhibited the highest spikelet fertility rate in both years, while V1, V2, and V4 showed similar performance.

### 3.2. Effect of formulated tillering fertilizer on tiller-bud length and fresh weight

In 2022, at 5, 10, and 15 d after treatment, the tiller-bud length and fresh weight under treatment T1 were significantly higher than those under CK (Table 1). Specifically, tiller-bud length increased by 36.67%, 42.97%, and 23.56%, respectively, whereas fresh weight increased by 76.46%, 56.43%, and 16.45%, respectively. At 5 d, the tiller-bud length and fresh weight of variety V3 were 103.40%, 62.53%, 46.74%, 140.37%, 45.92%, and 70.01% higher, respectively, than those of the other three varieties. At 10 d, variety V4 showed significantly greater tiller-bud length and fresh weight than the other three varieties, with increases of 96.22%, 41.09%, and 10.48% in length and 133.48%, 59.69%, and 12.16%, respectively. In contrast, at 15 d, the tiller-bud length and fresh weight of variety V2 were significantly lower than those of the other three varieties by 55.79%, 10.48%, and 70.27%, and 50.89%, 73.42%, and 73.42%, respectively. In 2023, similar to the 2022 results, both the tiller-bud length and fresh weight under T1 were significantly greater than those under CK. At the early tillering stage, V4 exhibited the most significant increase in tiller-bud length, which was significantly greater than that of the other three varieties. These findings indicate that the formulated tillering fertilizer can significantly promote tiller bud growth. Moreover, there were highly significant differences in tiller-bud length and fresh weight between varieties and treatments, and in the interaction effects between varieties and treatments.

**Table 1:** Effects of Different Tillering Fertilizers on Tiller Bud Length and Fresh Weight.

Year	variety	Treatment	Tiller Bud Length/cm ($\overset{―}{\text{x}}$_10_)			Tiller Bud Fresh Weight/mg ($\overset{―}{\text{x}}$_10_)		
			5 d	10 d	15 d	5 d	10 d	15 d
2022	V1	CK	1.27b	0.84b	1.63b	50.33b	47.33b	106.67b
		T1	3.92a	2.78a	2.89a	276.67a	185.67a	156.67a
	V2	CK	2.56b	2.40b	0.92b	171.33b	168.67b	57.00b
		T1	3.93a	2.63a	1.08a	367.33a	172.00a	72.33a
	V3	CK	5.08b	2.95b	3.12b	324.00b	179.67b	233.33b
		T1	5.48a	3.47a	3.40a	462.00a	305.33a	253.33a
	V4	CK	3.52a	2.93b	3.18b	219.00b	229.33b	215.00b
		T1	3.67a	4.16a	3.55a	243.33a	314.67a	230.33a
	*F* Value	V	1859.46**	1909.69**	6880.03**	2739.10**	1622.83**	1710.93**
		T	1847.48**	3072.66**	1543.93**	6304.34**	2549.80**	157.52**
		V × T	456.60**	465.22**	359.56**	585.74**	304.17**	17.34**
2023	V1	CK	0.73b	0.84b	1.01b	23.57b	59.61b	95.98b
		T1	1.59a	1.64a	1.88a	91.11a	75.32a	111.40a
	V2	CK	0.80b	0.75b	0.83b	36.90b	70.98b	105.73b
		T1	1.04a	1.03a	1.83a	175.32a	205.11a	190.98a
	V3	CK	0.83b	1.41b	1.34b	47.19b	115.82b	109.53b
		T1	1.52a	1.62a	1.87a	107.03a	256.94a	176.61a
	V4	CK	1.06b	1.54b	1.66b	33.11b	116.98b	125.44b
		T1	1.65a	1.77a	1.95a	103.53a	126.23a	287.86a
	*F* Value	V	65.44**	458.46**	251.58**	639.42**	1819.22**	2903.32**
		T	724.17**	581.73**	2668.66**	10351.84**	4261.31**	10981.38**
		V × T	35.20**	80.71**	158.62**	488.49**	990.18**	1495.23**

Notes: Different letters indicate significant differences among treatments at p < 0.05. * and **mean significance at the 0.05 and 0.01 probability levels, respectively. x_10_:denotes the average of 10 tiller buds; same notation applies hereafter

A comparison between years revealed that tiller bud growth under all treatments was generally better in 2023 than in 2022. Among varieties, V3 and V4 showed more obvious advantages in tiller bud growth, while V1 and V2 were relatively weaker. Moreover, the variety × treatment (V × T) interaction effects were all highly significant, indicating genetic differences among varieties.

### 3.3. Effects of the formulated tillering fertilizer on hormone contents in rice tiller buds

**Fig 3:**
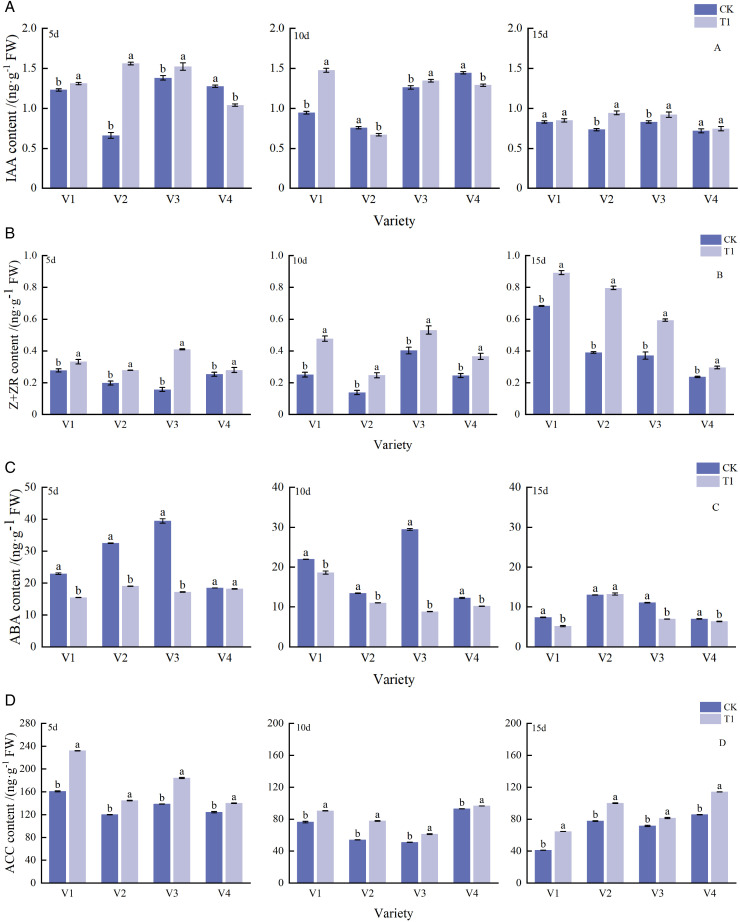
Effects of different tillering fertilizers on hormone contents in tiller buds. Note: Different letters indicate significant differences (*p* < 0.05) among treatments within the same variety; the same notation is used hereafter.

Analysis of auxin (IAA) content in tiller buds at 5, 10, and 15 d during the early tillering stage in 2022 (Fig 3A) revealed that Kecha fertilizer significantly increased IAA content, with an average increase of 13.00% compared with the control (CK). Significant interaction effects were observed among variety (V), treatment (T), and their interactions (V × T). Under treatment T1, the IAA contents of V1 and V3 at 5, 10, and 15 d were significantly higher than those of CK, with respective increases of 6.40%, 56.03%, and 2.49% (V1) and 10.30%, 6.80%, and 10.98% (V3). For V2, the IAA content in T1 was significantly higher than that in CK at 5 and 15 d (by 135.60% and 28.34%, respectively), whereas no significant difference was detected at 10 d. In the case of V4, the IAA level under T1 was significantly higher than that under CK at day 15, but significantly lower at days 5 and 10.

During the development of rice tiller buds, Kecha fertilizer significantly increased the content of zeatin + zeatin riboside (Z + ZR), with an average increase of 52.23% compared to CK, and significant interaction effects were found among V, T, and V × T (Fig 3B). The Z + ZR content peaked at 15 d during the early tillering stage, with V1 showing significantly higher levels than the other three varieties. After 15 d of T1 treatment, the Z + ZR levels of all four varieties were significantly higher than those of CK, by 30.64%, 104.02%, 59.93%, and 25.20%, respectively. At 5 and 10 d following the T1 treatment, V3 exhibited the highest Z + ZR content, which was 161.49% and 31.92% higher than that of CK, respectively.

When Kecha fertilizer was applied, the abscisic acid (ABA) content in tiller buds was significantly lower than that in CK, showing a downward trend during growth, with an average decrease of 30.86% (Fig 3C). Significant interaction effects of V, T, and V × T were observed for the ABA content. At 15 d in the early tillering stage, V2 had a higher ABA content under T1 than under CK, accompanied by a sharp decline in bud length and fresh weight. This suggests that increased ABA content may inhibit tiller bud elongation. For the other varieties, the ABA content in T1 was significantly lower than that in the respective CK groups. In V3, ABA levels decreased dramatically at 10 and 15 d after fertilization, being 69.90% and 37.21% lower than those in CK, respectively.

During the early tillering stage, 1-aminocyclopropane-1-carboxylic acid (ACC) content exhibited significant interactions with V, T, and V × T (Fig 3D). Kecha fertilizer treatment resulted in an average increase of 26.05% in ACC content relative to CK. Under the T1 treatment, the ACC content of all varieties peaked at day 5 and was significantly higher than that of CK by 44.14%, 20.57%, 32.98%, and 12.70%, respectively. At 10 and 15 d under the T1 treatment, V4 had the highest ACC content, exceeding that of CK by 3.17% and 33.15%, respectively. Across all varieties, the ACC content reached its maximum on day 5, with V1 recording the highest value (44.14% higher than that of CK).

### 3.4. Effects of the formulated tillering fertilizer on the ratios of endogenous IAA/(Z + ZR) and ABA/(Z + ZR) in rice tiller buds

During the vigorous tillering stage, the IAA/(Z + ZR) and ABA/(Z + ZR) ratios in tiller buds under the T1 treatment were markedly lower than those in CK, showing a continuous decrease as the buds developed, with average reductions of 27.64% and 55.30%, respectively(Table 2). For V3, the IAA/(Z + ZR) and ABA/(Z + ZR) ratios were highest at 5 d in the CK treatment but declined sharply under the T1 treatment by 58.04% and 83.40%, respectively. In V1, the ratios of IAA/(Z + ZR) and ABA/(Z + ZR) reached their lowest levels at 15 d under the T1 treatment, being 21.55% and 46.23% lower than in CK, respectively. Other varieties also exhibited a significant decrease in the IAA/(Z + ZR) ratio under the T1 treatment. These findings suggest that the formulated tillering fertilizers promote tiller bud initiation and growth.

**Table 2:** Effects on the Values of IAA/(Z + ZR) and ABA/(Z + ZR) in Rice Tiller Buds.

variety	Treatment	IAA/(Z + ZR)			ABA/(Z + ZR)		
		5d	10d	15d	5d	10d	15d
V1	CK	4.45a	3.79a	1.22a	82.93a	88.02a	10.87a
	T1	3.96a	3.10b	0.96b	46.75b	39.08b	5.85b
V2	CK	3.37b	5.57a	1.89a	165.00a	98.73a	33.53a
	T1	5.61a	2.73b	1.19b	68.64b	45.06b	16.69b
V3	CK	8.86a	3.14a	2.25a	252.95a	73.38a	30.16a
	T1	3.72b	2.54b	1.56b	41.98b	16.74b	11.82b
V4	CK	5.05a	5.92a	3.06a	73.16a	50.38a	29.77a
	T1	3.74b	3.54b	2.53b	65.44a	28.03b	21.75b
*F* Value	V	41.61**	42.04**	565.13**	120.99**	72.13**	750.79**
	T	60.92**	164.58**	321.51**	593.65**	631.02**	1672.79**
	V × T	102.76**	20.51**	11.26**	156.05**	18.85**	122.91**

Notes: Different letters indicate significant differences among treatments at p < 0.05. * and **mean significance at the 0.05 and 0.01 probability levels, respectively.

### 3.5. Effects of the formulated tillering fertilizer on Ca² ⁺ -Mg² ⁺ -ATPase activity in rice leaves

**Fig 4:**
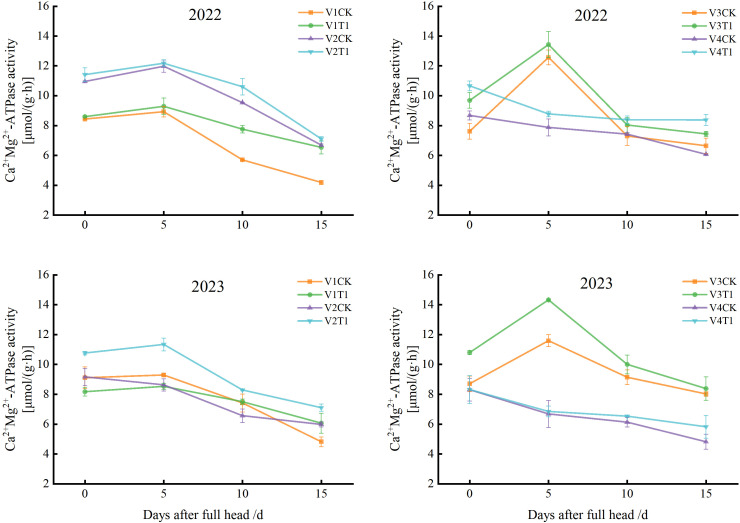
Effects of different tillering fertilizers on Ca² ⁺ -Mg² ⁺ -ATPase activity in leaves.

In 2022, significant differences in leaf Ca² ⁺ -Mg² ⁺ -ATPase activity were observed among the four rice varieties following treatment with the formulated tillering fertilizer. In varieties V1 and V2, leaf Ca² ⁺ -Mg² ⁺ -ATPase activity exhibited a rise-fall pattern (Fig 4), increasing from 0 to 5 days after heading and peaking at day 5, then declining between 5 and 15 days. However, enzyme activity under the T1 treatment remained higher than that under CK for 5–15 days. In V3, leaf Ca² ⁺ -Mg² ⁺ -ATPase activity under T1 treatment increased significantly by 14.51% relative to CK within 0–5 d after heading, peaking at 13.44 μmol/(g·h) at day 5, which was 1.07 times higher than that of CK. In 2023, significant variations in leaf Ca² ⁺ -Mg² ⁺ -ATPase activity were again detected among the four rice varieties after heading. In V1, V2, and V3, leaf Ca² ⁺ -Mg² ⁺ -ATPase activity followed a similar increasing-decreasing trend, rising from 0–5 d after heading, peaking at day 5, and declining from 5 to 15 d. All varieties exhibited higher leaf Ca² ⁺ -Mg² ⁺ -ATPase activity than CK between 5 and 15 d following fertilization, except for V1 under T1 treatment, which showed slightly lower values than CK from 5–10 d. Specifically, in V2 and V3, the maximum leaf Ca² ⁺ -Mg² ⁺ -ATPase activities under T1 treatment were recorded at 5 d after heading, reaching 11.35 μmol/(g·h) and 14.34 μmol/(g·h), respectively. For V4, the overall two-year trend showed a gradual decline in enzyme activity, yet values under the T1 treatment remained consistently higher than those under CK.

### 3.6. Effects of the formulated tillering fertilizer on the yield and contribution rates of the main stem and different tiller orders

As shown in Table 3, the yield and contribution rates of the main stem, primary tillers, and secondary tillers differed significantly among the varieties, treatments, and their interactions. In 2022, the yields of the main stem, primary tillers, and secondary tillers under the T1 treatment are 6.96%, 18.42%, and 8.86% higher than those under the CK treatment, respectively. In the T1 treatment, the contribution rates of the main stem and secondary tillers to the total yield were lower than those in the CK treatment, whereas the contribution rate of primary tillers was significantly higher. The V3 variety had the highest mainstem yield, primary tiller yield, and contribution rate, whereas V1 and V2 exhibited significantly higher secondary tiller yields and contribution rates than the other two varieties. Under the V3T1 treatment, the highest main stem and primary tiller yields were recorded, contributing 9.79% and 56.63% of total yield, respectively. Under the V2T1 treatment, secondary tillers achieved the highest yield, contributing 39.74% of the total yield.

**Table 3:** Effects of Different Tillering Fertilizers on Yield and Contribution Rate of Main Stem and Different Tiller Orders.

Year	variety	Treatment	Yield(kg/hm^2^)			Contribution Rate(%)		
			Main Stem	Primary Tiller	secondary tiller	Main Stem	Primary Tiller	secondary tiller
2022	V1	CK	760.51b	4136.90b	3306.79b	9.07a	51.49b	39.44a
		T1	815.70a	5436.51a	3546.84a	8.40b	55.07a	36.53b
	V2	CK	874.42a	4468.48b	3204.52b	10.02a	53.26a	36.72b
		T1	864.10a	4385.50a	3759.40a	9.14b	51.12b	39.74a
	V3	CK	885.03b	4468.29b	2653.86b	10.81a	56.77a	32.42a
		T1	944.35a	5642.89a	3239.68a	9.79b	56.63a	33.58a
	V4	CK	779.92b	4518.28b	3381.59a	9.38a	49.99b	40.63a
		T1	905.24a	5040.91a	3112.64b	9.61a	57.34a	33.05b
	*F* Value	V	49.83**	48.49**	58.76**	44.32**	73.97**	75.98**
		T	58.67**	1645.88**	78.09**	37.05**	92.70**	32.33**
		V × T	13.67**	127.61**	39.76**	8.57**	87.40**	72.05**
2023	V1	CK	786.78a	4370.33a	2834.81a	9.84a	54.69a	35.47a
		T1	833.53a	4400.11a	2931.57a	10.21a	53.89a	35.90a
	V2	CK	927.95a	4452.42b	2902.10a	11.20a	53.75b	35.05a
		T1	862.52b	5120.91a	3022.22a	9.58b	56.86a	33.56a
	V3	CK	842.06a	3696.01b	2477.22a	12.00a	52.68b	35.32a
		T1	789.42b	4128.83a	2397.08a	10.79b	56.44a	32.77b
	V4	CK	805.55b	3878.89b	3667.95b	9.64a	46.44a	43.91a
		T1	988.49a	5432.55a	5050.10a	8.62b	47.36a	44.02a
	*F* Value	V	16.25**	73.33**	421.58**	61.82**	84.81**	114.72**
		T	5.48*	221.22**	87.85**	53.92**	16.75**	3.94
		V × T	23.19**	51.03**	69.24**	13.010**	5.99**	2.54

Notes: Different letters indicate significant differences among treatments at p < 0.05. * and **mean significance at the 0.05 and 0.01 probability levels, respectively.

In 2023, the yields of the main stem, primary tillers, and secondary tillers under the T1 treatment exceeded those of CK across all varieties. The contribution rate of primary tillers was the highest under the T1 treatment, while that of secondary tillers was lower than that of CK. In variety V4, the main stem, primary tillers, and secondary tillers under T1 treatment achieved the highest yields of 988.49 kg/hm², 5,432.55 kg/hm², and 5,050.10 kg/hm², respectively. This suggests that, compared with other varieties, V4 exhibits a greater yield enhancement potential when treated with the formulated tillering fertilizer.

A comparison between the two years showed that the grain yield under all treatments was generally higher in 2022 than in 2023, and the variety × treatment (V × T) interaction effects all reached a significant level. The regulatory effects of fertilization treatments on grain yield and its contribution rate were more pronounced in 2022, with T1 exhibiting a greater yield increase and a more distinct differentiation in contribution rate relative to CK. In 2023, although the treatment effects remained significant, both the yield increase amplitude and the differences in contribution rate were reduced. Among the tested varieties, V3 and V4 consistently presented higher yield and contribution rate of primary tillers in both years, whereas V1 and V2 performed relatively poorly in these aspects.

## 4. Discussion

### 4.1. Impact of formulated tiller fertilizer on the growth and development of machine-transplanted rice

Previous research has demonstrated that compound fertilizers effectively enhance material transformation processes in rice, boosting dry matter translocation and its rate, which subsequently increases the harvest index [19]. Optimizing nitrogen management can reduce the duration of the rice establishment (green-up) period, stimulate the formation of productive tillers, facilitate the accumulation of canopy assimilates, and expand photosynthetic leaf area, thereby improving light-use efficiency during the reproductive growth stage [20]. The application of Si and Zn fertilizers is known to accelerate early tiller development, increase stem/tiller count, improve leaf area index (LAI), and consequently promote biomass accumulation [21,22]. Moreover, the use of Si, Zn, and Mg fertilizers consistently increases the number of effective tillers and panicles, elevates photosynthetic efficiency metrics, and significantly boosts yield by enhancing the photoassimilation flux of the canopy [23,24]. Organosilicon high-tower compound fertilizers promote rice tillering and improve the rice harvest index by simultaneously increasing the effective panicle number and optimizing assimilate transport, improving the rice harvest index [19]. Our current study found that applying the formulated tiller fertilizer effectively enhanced the total number of stems and tillers and increased the panicle formation rate, suggesting that the secondary and micronutrients present in the formulated fertilizer are capable of promoting the early and vigorous development of tillers, thus increasing both effective tillers and panicles, which is consistent with the existing literature.

Studies have indicated that the dormancy and growth of crop lateral buds or branches are governed by their endogenous hormones, which are primarily constrained by the levels and balance of hormones, including CTK, ABA, and auxins. Zinc, as a core cofactor of tryptophan aminotransferase, flavin monooxygenase and isopentenyl transferase, governs rice tillering by precisely regulating the CTK/IAA ratio in tiller buds. Under zinc deficiency, the activities of tryptophan aminotransferase and flavin monooxygenase decrease, leading to impaired IAA synthesis and disordered polar transport, which inhibits the germination of tiller buds [25,26]. Zn facilitates the synthesis and transport of indole-3-acetic acid (IAA) and stimulates CTK biosynthesis within rice plants. Because of this synergistic effect, an elevated ratio of CTK to IAA in the tiller buds can drive the growth of rice tiller buds [27]. Silicon can up-regulate the expression of IPT genes to enhance cytokinin (CTK) synthesis, reduce the polar transport and bud accumulation of IAA, and increase the CTK/IAA ratio to break the dormancy of tiller buds. Meanwhile, it inhibits the signaling of gibberellin (GA) and strigolactone (SL), and synergistically promotes the germination and elongation of tiller buds in late-transplanted rice [28]. Our research showed that the formulated tiller fertilizer (T1) treatmentsignificantly increased the content of IAA/Z + ZR, and ACC in the buds, while simultaneously reducing ABA content in the buds. Over the two-year period, the T1 treatment resulted in significantly greater bud length and fresh weight of tiller buds compared to the control, indicating that the secondary and micronutrients applied via the formulated tiller fertilizer can alter endogenous hormone content, consequently promoting tiller bud growth and vigorous early onset of tillering. Specifically, the effective panicle rates for Taifengyou 208 and Nei 6 You 6368 were higher in the CK treatment than in the T1 treatment. This phenomenon may stem from the higher ABA content in the buds under the control treatment, which effectively suppressed non-productive tillers originating from the upper nodes, thereby increasing the number of productive tillers, a finding consistent with that of a previous study [29].

Rice leaves serve as core sites for the generation of photosynthetic products. The degree of photoassimilate accumulation dictates the magnitude of rice grain yield and is fundamental for dry matter accumulation and yield formation [30]. Late seedling transplanting leads to a prolonged regreening period, a sharp shortening of the tillering stage, delayed construction of photosynthetic leaf area and reduced accumulation of canopy assimilates. The grain filling stage is compressed, and ineffective tillers consume excessive photosynthates, which exacerbates the imbalance between source and sink supply and demand. Zn is known to elevate chlorophyll content in rice plants, catalyze the photochemical reactions of chlorophyll, and stimulate carbohydrate synthesis and metabolism, thus ameliorating the insufficient photosynthesis in rice caused by late transplanting [31]. Regulation via Ca–Mg–PO4 compound fertilizer works by optimizing the effective panicle rate, increasing rice dry matter accumulation and the relative chlorophyll content of the flag leaf, thereby establishing a highly efficient synergistic mechanism for “Source-Sink” metabolic flow [32]. Si application has been shown to enhance chlorophyll concentration, light-use efficiency, ATP concentration, and CO2 assimilation [33]. The results of this study indicated that the application of formulated tillering fertilizer could significantly enhance the ATPase activity of late-transplanted rice, with significant differences observed among different varieties. This indicates that multiple micronutrients, such as Zn and Mg, present in the formulated tiller fertilizer likely participate in regulating photosynthesis, respiration, and other essential plant metabolic processes, thereby ensuring that rice leaves possess a high production capacity and material accumulation levels during the late growth phase, optimizing nutrient supply to satisfy the demand for materials during grain filling, and ultimately ensuring an increase in rice yield.

### 4.2. Impact of formulated tiller fertilizer on the yield of machine-transplanted rice

Rice yield is determined by a combination of effective panicles per unit area, spikelets per panicle, 1000-grain weight, and grain-filling rate, where effective panicles and spikelets per panicle are the core regulatory factors for enhancing yield [34]. Late seedling transplanting hinders the early occurrence of tillering and reduces the number of effective panicles. It shortens the young panicle differentiation stage, leading to a decrease in the number of grains per panicle, and insufficient grain filling causes varying degrees of reduction in seed setting rate and 1000-grain weight. Overall, the yield is reduced by 10% to 30% compared with timely transplanting [17]. Fertilization can regulate rice yield, and elements such as zinc, magnesium and silicon are crucial to its growth [35]. The application of secondary and micronutrient fertilizers, including Si, Zn, and boron, led to significant improvements in rice spikelets per panicle, 1000-grain mass, and grain filling rate [36–38]. Formulated fertilization had a notable effect on rice growth and yield composition, with grain yield showing a significant or highly significant positive correlation with effective panicles, spikelets per panicle, and 1000-grain weight [39]. Moderate Zn fertilizer applications can increase the number of productive rice tillers while reducing non-productive tiller formation, thereby enhancing rice yield [40].

Our study showed that application of the formulated tiller fertilizer significantly boosted rice yield, primarily by enhancing the emergence of primary tillers, It forms a dominant system for nutrient absorption and utilization with the main stem, where photosynthetic products and mineral nutrients are largely absorbed by the main stem and primary tillers, reducing the number of secondary tillers and thereby promoting rice yield increase. This finding is consistent with the research of Lei Xiaolong [41].

Certainly, crop phenotypes are jointly influenced by genotype and environment, and the superior performance of the fertilizers and varieties observed in this study may be associated with the climatic or soil conditions of the experimental site. Therefore, to further verify the stability of the results and confirm their applicability under different ecological environments, subsequent multi-year and multi-location validation experiments need to be conducted across multiple growing seasons and ecological regions. Meanwhile, single-factor control experiments should be set up, along with the determination of soil trace element contents and the measurement of hormone levels and enzyme activities in multiple plant tissues.

## 5. Conclusions

Compared with the single application of compound fertilizer, the combined application of medium and trace element fertilizers containing silicon, magnesium and zinc (1 kg/m²) exhibited a superior regulatory effect on late-transplanted rice in the hilly areas of northwest Sichuan, which could effectively alleviate the problems of overlong seedling age and yield reduction caused by late transplanting. This treatment significantly regulated the endogenous hormone contents in tiller buds within 15 days of tillering, promoted tiller bud growth and stem-tiller number establishment, and enhanced rice ATPase activity. Consequently, it notably increased the panicle formation rate and effective panicle number per unit area, ultimately achieving rice yield increase. Moreover, such regulatory effects were more prominent in the V3 and V4 varieties, indicating that these two varieties were well adapted to the above trace element fertilizer combination, which could serve as the optimal variety-fertilizer matching scheme for the high-yield cultivation of late-transplanted rice in the hilly areas of northwest Sichuan.

## Supporting information

S1 File The data obtained from the experiment are provided in the supporting information file “S experimental data”. (XLSX)
